# Rapid detection of colistin resistance in *Acinetobacter baumannii* using MALDI-TOF-based lipidomics on intact bacteria

**DOI:** 10.1038/s41598-018-35041-y

**Published:** 2018-11-15

**Authors:** Laurent Dortet, Anais Potron, Rémy A. Bonnin, Patrick Plesiat, Thierry Naas, Alain Filloux, Gerald Larrouy-Maumus

**Affiliations:** 1 MRC Centre for Molecular Bacteriology and Infection, Department of Life Sciences, Faculty of Natural Sciences, Imperial College London, London, SW7 2AZ UK; 2 Department of Bacteriology- Hygiene, Bicêtre Hospital, Assistance Publique - Hôpitaux de Paris, Le Kremlin-Bicêtre, France; 3 EA7361 “Structure, dynamic, function and expression of broad spectrum β-lactamases”, Paris-Sud University, LabEx Lermit, Faculty of Medecine, Le Kremlin-Bicêtre, France; 4 French National Reference Center for Antibiotic Resistance, Le Kremlin-Bicêtre, France; 5 Bacteriology unit, University hospital of Besançon, Besançon, France

## Abstract

With the dissemination of extremely drug resistant bacteria, colistin is now considered as the last-resort therapy for the treatment of infection caused by Gram-negative bacilli (including carbapenemase producers). Unfortunately, the increase use of colistin has resulted in the emergence of resistance as well. In *A. baumannii*, colistin resistance is mostly caused by the addition of phosphoethanolamine to the lipid A through the action of a phosphoethanolamine transferase chromosomally-encoded by the *pmrC* gene, which is regulated by the two-component system PmrA/PmrB. In *A. baumannii* clinical isolate the main resistance mechanism to colistin involves mutations in *pmrA*, *pmrB* or *pmrC* genes leading to the overexpression of *pmrC*. Although, rapid detection of resistance is one of the key issues to improve the treatment of infected patient, detection of colistin resistance in *A. baumannii* still relies on MIC determination through microdilution, which is time-consuming (16–24 h). Here, we evaluated the performance of a recently described MALDI-TOF-based assay, the MALDIxin test, which allows the rapid detection of colistin resistance-related modifications to lipid A (*i.e* phosphoethanolamine addition). This test accurately detected all colistin-resistant *A. baumannii* isolates in less than 15 minutes, directly on intact bacteria with a very limited sample preparation prior MALDI-TOF analysis.

## Introduction

Currently, antimicrobial resistance is on top of the agenda for scientists and governments, while pan-resistant organisms are fast emerging. Any lack of swift commitment and action to improve diagnostic and prevention would irremediably take us back to the dark age of dreadful and devastating epidemics. The pipeline of new antibiotics is very limited, and colistin is now considered as the last resort therapy for the treatment of infection caused by multidrug resistant (MDR) Gram-negative bacteria, such as carbapenemase-producing *Acinetobacter baumannii*^1^. Unfortunately, with an increase in the use of colistin to treat carbapenem-resistant *Acinetobacter baumannii* infections, colistin resistance is emerging^2^.

In Gram-negative bacteria, acquired resistance to polymyxins results mostly from modifications of the drug target, *i.e*. the lipopolysaccharide (LPS). These modifications correspond to addition(s) of cationic groups such as 4-amino-L-arabinose (L-Ara4N) and/or phosphoethanolamine (pETN) on the lipid A, the anchor of the LPS. Unlike *Enterobacteriaceae*, *A. baumannii* lacks all the genes required for L-Ara4N biosynthesis. Accordingly, colistin resistance is caused by the addition of pETN to the lipid A on position 1 or 4’ by an EptA-like phosphoethanolamine transferase chromosomally-encoded by the *pmrC* gene. As well, mutations in the chromosome-encoded *pmrA* and *pmrB* genes result in a constitutive activation of the PmrA/PmrB two-component system, which in turn upregulates the expression of *pmrC*^3–5^. Recently, plasmid-mediated resistance to polymyxin, named MCR, was described in *Enterobacteriaceae*. The *mcr* genes (*mcr-1*, *-2*, *-3*, *-4*, *-5, -6, -7* and *-8*) also encode a phosphoethanolamine transferase involved in addition of pETN on the lipid A^6^. Although *mcr* genes were found to have disseminated in *Enterobacteriaceae* (mostly *Escherichia coli*), they are not currently reported in *Acinetobacter* for which resistance to polymyxin is still restricted to chromosome-encoded resistance^5–7^.

Rapid detection of resistance is one of the key issues to improve the treatment of patient infected with MDR bacteria. However, detection of colistin resistance in *Acinetobacter* relies on minimal inhibition concentration determination using broth microdilution which is the gold standard for polymyxins susceptibility testing^8^. This method has been chosen as the unique reference by The Clinical Laboratory Standard Institute (CLSI) and by the European Committee on Antimicrobial Susceptibility Testing (EUCAST), recently gathered in a joint subcommittee^9^, which ruled out methods classically used for determination of antimicrobial susceptibility, such as agar dilution, disk diffusion, gradient diffusion (Etest) and automated systems (MicroScan^®^, Vitek^®^ 2, BD Phoenix^™^) for which high rates of false susceptibility were observed. Consequently, results can only be obtained up to 24 h after bacterial isolation^10^. Recently, a biochemical test, the rapid polymyxin NP test, that detects bacterial growth in the presence of a defined concentration of a polymyxin (3.5 mg/L) has been developed^11^. With this colorimetric test, the bacterial growth, or absence of, is tracked on the basis of carbohydrate metabolism^12^. The acid formation resulting from carbohydrate metabolism during the continuous growth of colistin resistant bacteria even in presence of polymyxin, is detected by the color change of a pH indicator. Also this test was validated for fermenting bacteria, such as *Enterobacteriaceae*, it cannot conceptually be applied to non-fermenters such as *Acinetobacter*.

Recently, we developed a rapid technique using MALDI-TOF able to detect colistin resistance directly on intact bacteria in less than 15 minutes, the MALDIxin test^13^. This cutting-edge method has been validated for *E. coli* for which it can detect not only polymyxin resistance but also discriminated chromosome- and plasmid-encoded resistance (i.e. *mcr*). This method is based on the MALDI-TOF on-target extraction of the free lipid A directly from bacterial colonies^14^. Indeed, this technique does not required prior time-consuming processes for the whole extraction of the LPS and hydrolysis into its lipid A using highly hazardous reagents^14^. In this study, the peaks corresponding to pETN-modified Lipid A are detected in all colistin-resistant *E. coli* isolates^13^. In addition, a specific peak corresponding to the addition of pETN associated with dephosphorylation of the native lipid A was found only in MCR-producing *E. coli* strains^13^.

Here, we evaluated the ability of the MALDIxin test to detect colistin-resistance in *A. baumannii*.

## Results and Discussion

**Figure 1:**
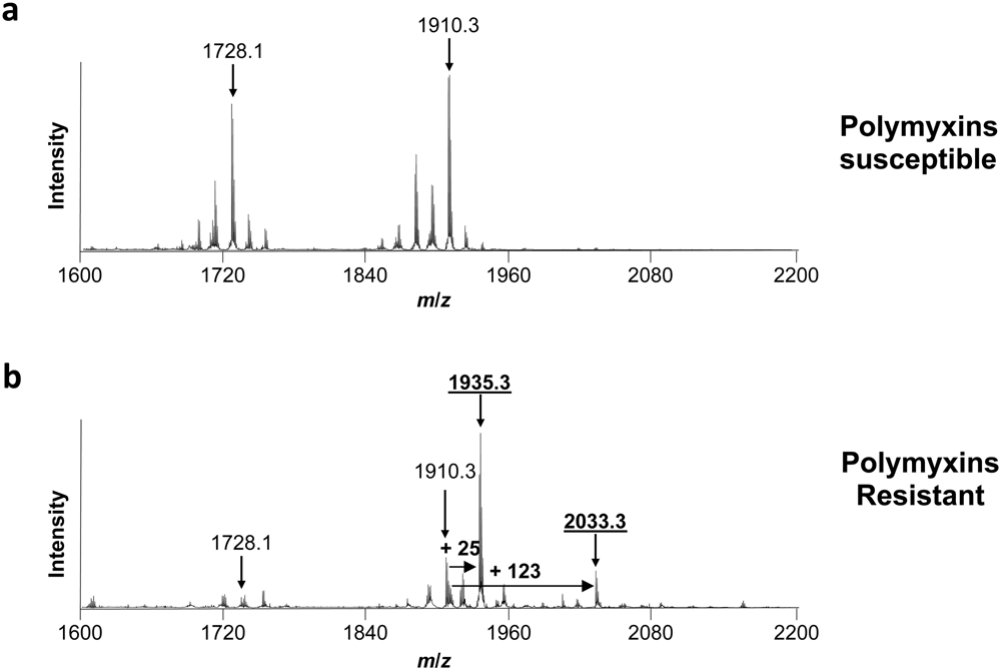
Results of the MALDIxin test on *A. baumannii*. Representative spectra of a polymyxin-susceptible *A. baumannii* isolate (Ab-S1) (panel a) and a colistin-resistant *A. baumannii* isolate (Ab-R1) (panel b). Peaks of interest are indicated. The peaks at *m/z* 1728.1 *m/z* and 1910.3 *m/z* corresponds to the native lipid A of *A. baumannii*, the peak at *m/z* 1935.3 likely corresponds to the addition of pETN on the phosphate group at position 4’ of the native lipid A of *A. baumannii* with concomitant loss of the phosphate group on position 1, and the peak at *m/z* 2033.3 corresponds to the addition of one pETN on the phosphate group at position 1 of the native lipid A of *A. baumannii*.

In polymyxin susceptible *Acinetobacter baumannii*, the mass spectrum is dominated by 2 set of peaks centred at *m*/*z* 1728.1 and *m*/*z* 1910.3 (Fig. 1a), assigned to bis-phosphorylated hexa-acyl and bis-phosphorylated hepta-acyl lipid A, with acyl chain ranging from 12 to 14 carbons in length, respectively^15,16^. In colistin resistant strains, the mass spectrum is dominated by 2 set of peaks centred at *m*/*z* 1935.3 and *m*/*z* 2033.3, corresponding to the previously observed *m/z* +25 and *m/z* +123 shifts of mass unit of the native bis-phosphorylated hepta-acyl lipid A at *m*/*z* 1910.3 (Fig. 1b). These peaks were assigned to pETN-modified-bis-phosphorylated hepta-acyl lipid A with acyl chain of 12 carbons in length (*m*/*z* 2033.3)^15^, and pETN-modified-mono-phosphorylated hepta-acyl lipid A with acyl chain of 12 carbons in length (*m*/*z* 1935.3)^13^, As previously observed with MCR-producing *E. coli*, the addition of a pETN moiety onto the phosphate group at position 1 lead to a global shift of +123 *m*/*z* of the peak corresponding to the native lipid A, while the addition of a pETN moiety onto the 4′ of native lipid A is concomitant with loss of the phosphate group on position 1 leading to a global shift of +25 *m*/*z* of the peak corresponding to the native lipid A^13^. Since EptA-like and MCR-like enzymes are both phosphoethanolamine transferases, we expected to observe the appearance of two peaks at +25 *m*/*z* and +123 *m*/*z* related to pETN modified lipid A compare to the native lipid A, as it was previously shown with all MCR-producing *E. coli*^13^. Of note, these two specific peaks at *m*/*z* 1935.3 and *m*/*z* 2033.3 were also observed for the three resistant isolates for which the molecular mechanism remains unknown (Ab-R2, Ab-R4 and Ab-R6) (Fig. 1b, Table 1). However, although no mutation in *pmrA*, *pmrB* and *pmrC* was identified, overexpression of the *pmrC*/*eptA* gene (encoding the pETN transferase) was systematically observed in these three isolates using qRT-PCR. As shown in Table 1 the peaks corresponding to the pETN modified lipid A (*m*/*z* 1935.3 and *m*/*z* 2033.3) were not observed with colistin susceptible isolates, while they were systematically in all colistin resistant isolates.

**Table 1:** Characteristics and results of the MALDIXin test on *Acinetobacter baumannii* isolates.

Name	Colistin MIC (mg/L)	Mechanism of colistin resistance	Acquired resistance to β-lactams^a^	Observed peaks			
				*m/*z 1728	*m/*z 1910	*m/*z 1935	*m/*z 2033
Ab-R1*	>64	Duplication of PmrB transmembrane domain	**OXA-23**	+	+	+	+
Ab-R2	>64	Unknown^b^	**OXA-23** + Case	+	+	+	+
Ab-R3	16	Mutation in PmrB (A226T)	**OXA-72** + Case	+	+	+	+
Ab-R4	>64	Unknown^b^	**OXA-23** + Case	+	+	+	+
Ab-R6	>64	Unknown^b^	**OXA-23** + Case + TEM-1	+	+	+	+
Ab-R5	4	Mutation in PmrB (A226V)	**OXA-23** + Case + TEM-1	+	+	+	+
Ab-R7	>64	Mutation in PmrB (R263H)	**OXA-23** + Case + TEM-1	+	+	+	+
Ab-R8**	64	Mutation PmrA (M12K)	WT	+	+	+	+
Ab-R12***	8	Mutation PmrB (A227V)	**OXA-23** + Case	+	+	+	+
Ab-S1^*^	0.5	−	**OXA-23**	+	+	−	−
Ab-S2	0.5	−	None	+	+	−	−
Ab-S3	≤0.25	−	**OXA-24/40** + CTX-M-115 + Case + TEM-1	+	+	−	−
Ab-S4	2	−	**OXA-23** + Case + TEM-1	+	+	−	−
Ab-S5	≤0.25	−	**NDM-1**	+	+	−	−
Ab-S6	≤0.25	−	Case	+	+	−	−
Ab-S7**	≤0.25	−	WT	+	+	−	−
Ab-S8***	0.5	−	**OXA-23** + Case	+	+	−	−

*Ab-R1 and Ab-S1 clinical isolates are isogenic^18^. **Ab-R8 (=AB CR17) and Ab-S7 (=AB CS01) are isogenic^19^. ***Ab-R12 and Ab-S8 are isogenic.

^a^Carbapenemases are bolded. Extended-spectrum β-lactamases are underlined. Case, overexpressed chromosome-encoded cephalosporinase. WT, wild-type.

^b^No mutation in *pmrA, pmrB* and *pmrC*. The expression of *pmrC* (verified by qRT-PCR) is increases of 172 ± 38 fold, 53 ± 5 fold and 108 ± 329 fold for Ab-R2, Ab-R4 and Ab-R6 isolates respectively compare to colistin susceptible Ab-S4.

Here, we demonstrated that the MALDIxin test is a rapid, accurate and cost effective technique based on MALDI-TOF for the detection of colistin resistance in *A. baumannii*. As previously described^13^, the routine use of the MALDIxin test will require switching the MALDI-TOF-MS machine to the negative ion mode due to the inherent negative charge of lipid A. This negative mode is not currently used for bacterial identification and thus it is locked in the MALDI-TOF MS machines commonly used in diagnostic. However, preliminary data indicate that MALDIxin test might be implemented on classical MALDI-TOF MS machines where this negative mode was unlocked (data not shown). In addition, one of the limitations of this study resides in the low number of tested isolates. However, the resistance mechanisms included in this study represent the most prevalent causes of colistin resistance in *A. baumannii* (mostly modification/mutation in PmrA or PmrB)^5,7,17^. Compared to the unique rapid detection method available for the detection of colistin resistance, the Polymyxin NP test, the MALDIxin test can work on a large panel of Gram negative bacteria, including non-fermenters (e.g. *A. baumannii*). In addition, this study paves the way for the future development of a rapid diagnostic test that could detect colistin resistance in all Gram-negative bacteria, including *Enterobacteriaceae* (*E. coli*, *K. pneumoniae*, *Salmonella*, …), *Acinetobacter* spp. and *Pseudomonas aeruginosa*. However, optimizations are still needed to allow the direct detection of L-Ara4N-modified lipid A which remains the main cause of colistin resistance in *K. pneumoniae* and *P. aeruginosa*.

## Methods

### Bacterial strains

A collection of 17 *A. baumannii* isolates including 9 colistin-resistant and 8 colistin-susceptible isolates were subjected to the MALDIxin test. The 9 polymyxin-resistant isolates, of which 5 harboured modifications of PmrB and one is mutated in PmrA (Table 1). For all colistin-resistant isolates, the modifications (mutation, deletion, disruption) in *phoP*, *phoQ*, *pmrA*, *pmrB* and *pmrC* were verified by whole genome sequencing (Illumina).

### Susceptibility testing

Colistin MIC was determined by broth microdilution according to the Clinical Laboratory Standard Institute (CLSI) and the European Committee on Antimicrobial Susceptibility Testing (EUCAST) guidelines^9^. Results were interpreted using EUCAST breakpoints as updated in 2018 (http://www.eucast.org/clinical_breakpoints/).

### MALDIxin test

The MALDIXin procedure was performed as previously described^13^. Briefly, a single colony cultured on Mueller-Hinton agar (bioMérieux, La Balme-les-Grottes, France) was resuspended in 200 μl of distilled water, washed three times with double distilled water and resuspended in 100 μl of double distilled water. 0.4 μL of the bacterial solution was loaded onto the target and immediately overlaid with 0.8 μL of a 2, 5-dihydroxybenzoic acid (DHB) matrix (Sigma Aldrich, Gillingham, United-Kingdom) used at a final concentration of 10 mg/mL in chloroform/methanol (CHCl_3_/MeOH) 90:10 v/v. Bacterial solution and matrix were mixed directly on the target by pipetting and the mix was dried gently under a stream of air (less than one minute). MALDI-TOF MS analysis was performed on a 4800 Proteomics Analyzer (Applied Biosystems, Foster City, USA) using the reflectron mode. Samples were analyzed by operating at 20 kV in the negative ion mode using an extraction delay time set at 20 ns. Mass spectrometry data were analyzed using Data Explorer version 4.9 (Applied Biosystems).

### Expression of pmrC/eptA

The three colistin-resistant *A. baumannii* that gave a positive signal for the presence of a peak related to the production of a phosphoethanolamine transferase activity, but were negative for modification in *pmrA*, *pmrB* and *pmrC* genes (Ab-R2, Ab-R4 and Ab-R6) were subjected to qRT-PCR to assess the expression of *pmrC*/*eptA* gene as previously described^16^. The expression of *pmrC/eptA* was normalized using 16 S RNA encoding gene. The fold change expression of *pmrC/eptA* was related to the basal expression of *pmrC*/*eptA* in the colistin susceptible Ab-S4 isolate.
